# Supplementary material for: A dissymmetric [Gd2] coordination molecular dimer hosting six addressable spin qubits
Source: Commun Chem. 2020 Nov 20;3:176. doi: 10.1038/s42004-020-00422-w (PMC9814487; doi:10.1038/s42004-020-00422-w)
Supplement: Supplementary file 1 — Supplementary Information [file 42004_2020_422_MOESM1_ESM.pdf]

Supporting Information for the manuscript:

A dissymmetric [Gd_2] coordination molecular dimer hosting six addressable spin qubits

Fernando Luis, Pablo J. Alonso, Olivier Roubeau, Verónica Velasco, David Zueco, David Aguilà, Jesús I. Martínez, Leoní A. Barrios, Guillem Aromí

Table of contents

Supplementary Tables

S. Table 1. Crystallographic and refinement parameters of [LaGd] and [GdLu] p. S2

S. Table 2. Details of H-bonding in [LaGd] and [GdLu] p. S3

S. Table 3. Coordination environment of the Gd sites in [LaGd] and [GdLu] p. S4

Supplementary Figures

S. Figure 1. H-bonding in the structures of [LaGd] and [GdLu] p. S5

S. Figure 2. Continuous shape measures for the Gd sites in [LaGd] and [GdLu] p. S6

S. Figure 3. CW-EPR spectra at X and Q bands for [LaGd], [GdLu] and [Gd_2] p. S7

S. Figure 4. Calculated transition and Rabi frequencies for resonant transitions in [LaGd] p. S8

S. Figure 5. Universality test for [Gd_2] p. S9

S. Figure 6A and B. Representative spin-echo decays for [LaGd], [GdLu] and [Gd_2] p. S10

S. Figure 7. Echo-induced EPR spectra from 2-pulse experiments and varying τ. p. S12

S. Figure 8. Echo-induced EPR spectra from 3-pulse experiments and varying T. p. S13

S. Figure 9. Echo-induced EPR spectra from sums over τ and T. p. S14

S. Figure 10 A, B and C. Fits of 2-pulse electron spin-echo decays p. S15

S. Figure 11 A, B and C. Fits of inversion recovery data p. S18

S. Figure 12. Field dependence of T_M , the ESE amplitude and the frequency of the modulation in the 2-p ESE decay for [LaGd], [GdLu] and [Gd_2] p. S21

S. Figure 13. Field dependence of T_1 and the inversion recovery amplitude for [LaGd], [GdLu] and [Gd_2] p. S22

S. Figure 14. Nutation experiments for [LaGd] p. S23

S. Figure 15. Nutation experiments for [GdLu] p. S24

S. Figure 16. Nutation experiments for [GdLu] p. S25

Supplementary Table 1. Crystal data for compounds [LaGd] and [GdLu].

Compound	[LaGd]	[GdLu]
Crystal size (mm^3)	0.86x0.18x0.12	0.40x0.03x0.03
Formula	$C_{50}H_{34}GdLaN_5O_{19}$, 5($C_5H_5N$), C_5H_6N	$C_{50}H_{34}GdLuN_5O_{19}$, 5($C_5H_5N$), C_5H_6N
FW (g mol^{-1})	1780.59	1816.65
Wavelength (Å)	0.71073	0.71073
Crystal system	monoclinic	monoclinic
Space group	$P2_1/n$	$P2_1/n$
Z	4	4
T (K)	100(2)	100(2)
a (Å)	14.4120(5)	14.268(2)
b (Å)	15.8616(6)	15.697(2)
c (Å)	32.9512(13)	33.048(4)
β (°)	90.6457(17)	92.267(5)
V (Å^3)	7532.1(5)	7395.8(17)
ρ_{calcd} (g cm^{-3})	1.570	1.631
μ (mm^{-1})	1.513	2.398
Independent reflections	17283 (R_{int} = 0.0407)	8238 (R_{int} = 0.1345)
restraints / parameters	507 / 1068	342 / 952
Goodness-of-fit on F^2	1.089	1.017
Final R_1 / wR_2 [I>2σ(I)]	0.0413 / 0.0501	0.0712 / 0.1564
Final R_1 / wR_2 [all data]	0.0903 / 0.0944	0.1150 / 0.1787
largest diff. peak and hole (e Å^3)	1.789 / –1.103	1.361 / –1.330

Supplementary Table 2. Details of H-bonding interactions in the structures of compounds [LaGd] and [GdLu].

D–H···A	D–H (Å)	H···A (Å)	D–A (Å)	D–H···A (º)
[LaGd]				
O19–H19B···N1S	0.895(19)	1.92(2)	2.810(5)	177(5)
O19–H19C···N2S	0.884(19)	1.98(3)	2.809(6)	156(4)
O5–H5···O4	0.84	1.83	2.564(4)	144.5
O10–H10···O9	0.84	1.80	2.540(4)	145.8
O15–H15···O14	0.84	1.89	2.610(4)	143.1
N3S–H3···O1	0.88	1.82	2.695(5)	176.1
[GdLu]				
O19–H19C···N1S	0.91(2)	1.90(3)	2.771(19)	161(10)
O19–H19D···N2S	0.90(2)	1.90(4)	2.71(2)	149(7)
O5–H5···O4	0.84	1.91	2.553(15)	132.5
O10–H10···O9	0.84	1.78	2.530(13)	148.1
O15–H15···O14	0.84	1.98	2.670(13)	152.1
N4S–H4SB···O2	0.88	1.92	2.73(2)	152.1

Supplementary Table 3. Metal–ligand bond distances (Å) and metal···metal separations (Å) in the structures of compounds [LaGd] and [GdLu].

[LaGd]		[GdLu]	
La1–O6	2.466(3)	Gd1–O14	2.355(10)
La1–O14	2.482(3)	Gd1–O6	2.380(10)
La1–O1	2.492(3)	Gd1–O1	2.390(10)
La1–O13	2.543(2)	Gd1–O16	2.409(11)
La1–O8	2.583(3)	Gd1–O13	2.415(10)
La1–O16	2.648(3)	Gd1–O8	2.470(10)
La1–N2	2.648(3)	Gd1–N2	2.500(12)
La1–O3	2.701(2)	Gd1–O3	2.582(9)
La1–N1	2.757(3)	Gd1–N1	2.598(11)
La1–O17	2.796(3)		
Gd2–O4	2.357(2)	Lu2–O4	2.288(10)
Gd2–O11	2.365(3)	Lu2–O8	2.300(10)
Gd2–O8	2.381(3)	Lu2–O11	2.317(10)
Gd2–O3	2.435(2)	Lu2–O3	2.357(9)
Gd2–O19	2.438(3)	Lu2–O19	2.376(10)
Gd2–O9	2.442(3)	Lu2–O9	2.385(10)
Gd2–O13	2.448(2)	Lu2–O13	2.407(9)
Gd2–N3	2.486(3)	Lu2–N3	2.430(13)
Gd2–N5	2.687(3)	Lu2–N5	2.638(13)
Gd2···La1	3.8941(3)	Gd1···Lu2	3.7610(11)

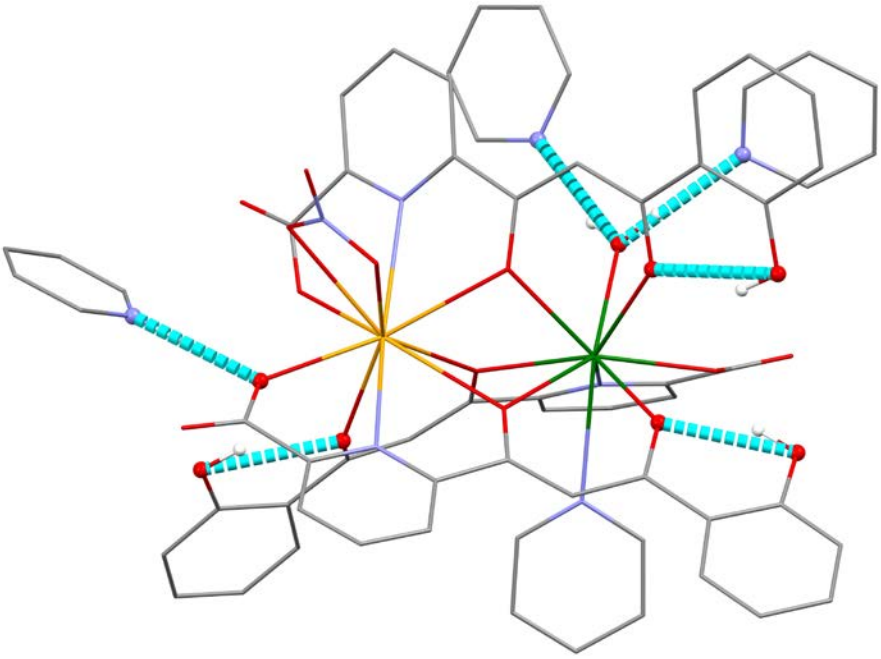

Supplementary Figure 1. H-bonding in the structure of [LaGd]. Similar interactions are present in the structure of [GdLu] (see Table S3). H-bonds are depicted as thick dashed light blue lines. Only atoms involved in H-bonds are shown as balls. Colour code: O, red; N, light blue; H, white; C, grey; La, orange; Gd, green.

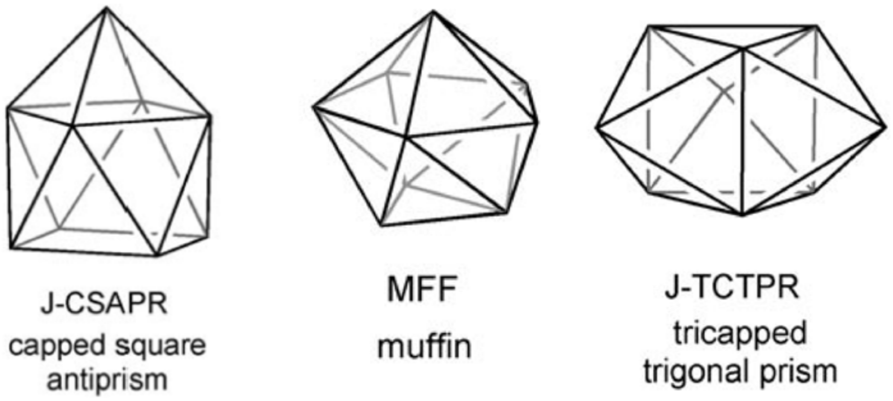

Supplementary Figure 2. Ideal nine-vertex polyhedral to which the coordination geometry of the Gd(III) ions in [LaGd], [GdLu] and [Gd_2] are compared through Continuous shape measures: capped square antiprism, muffin and tricapped trigonal prism. Calculated distances to these ideal polyhedral are:
Gd2 in [LaGd]: 0.657 to TCTPR, 0.701 to CSAPR and 1.223 to MFF
Gd1 in [GdLu]: 3.010 to MFF, 4.132 to CSAPR and 4.782 to TCTPR

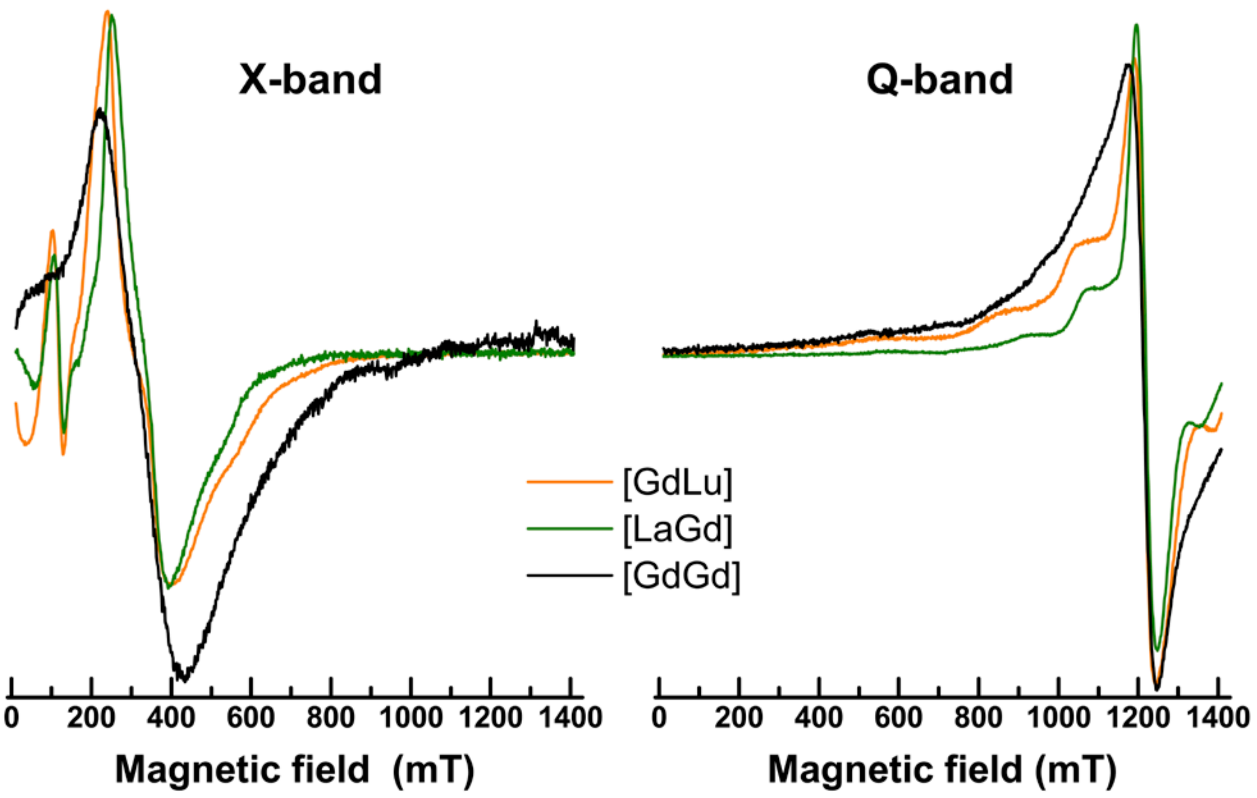

Supplementary Figure 3. X-band and Q-band cw-ESR spectra of polycrystalline samples of [LaGd], [GdLu] and [Gd_2] as indicated. The positions of the absorption derivative maxima and minima show that spectra measured on the latter compound are not simple superpositions of those measured on the former two, the difference being more visible at lower magnetic fields (X-band).

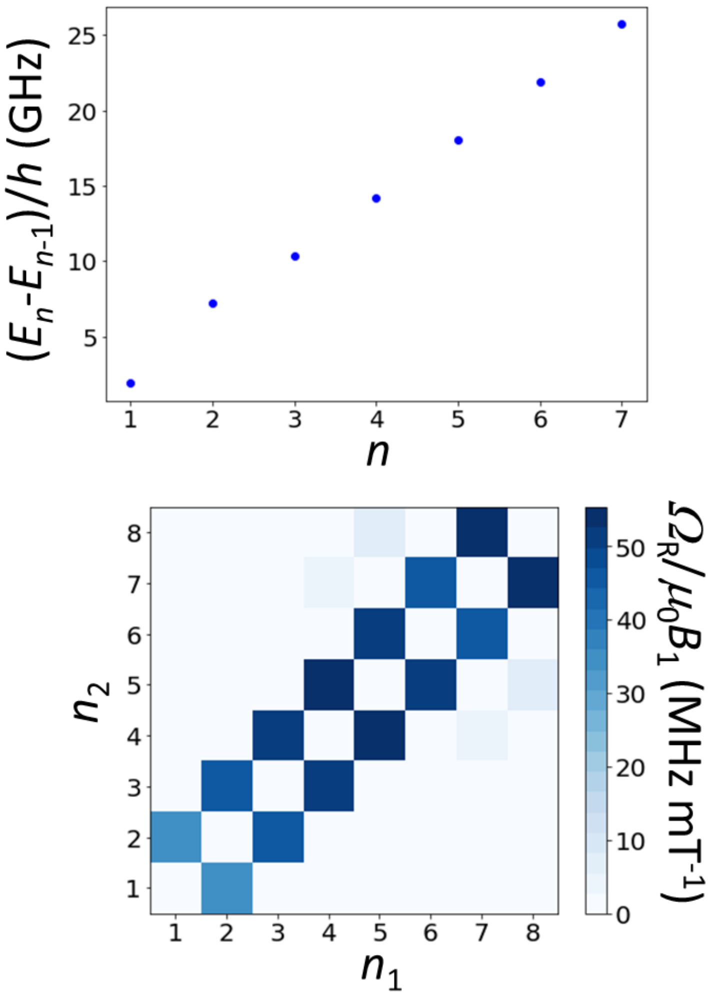

Supplementary Figure 4. Top: Transition frequencies between adjacent levels n-1 and n of [LaGd] calculated for a 0.5 T magnetic field applied along the z axis (easy axis). Bottom: Rabi frequencies for resonant transitions between different spin states of [LaGd], calculated at the same field.

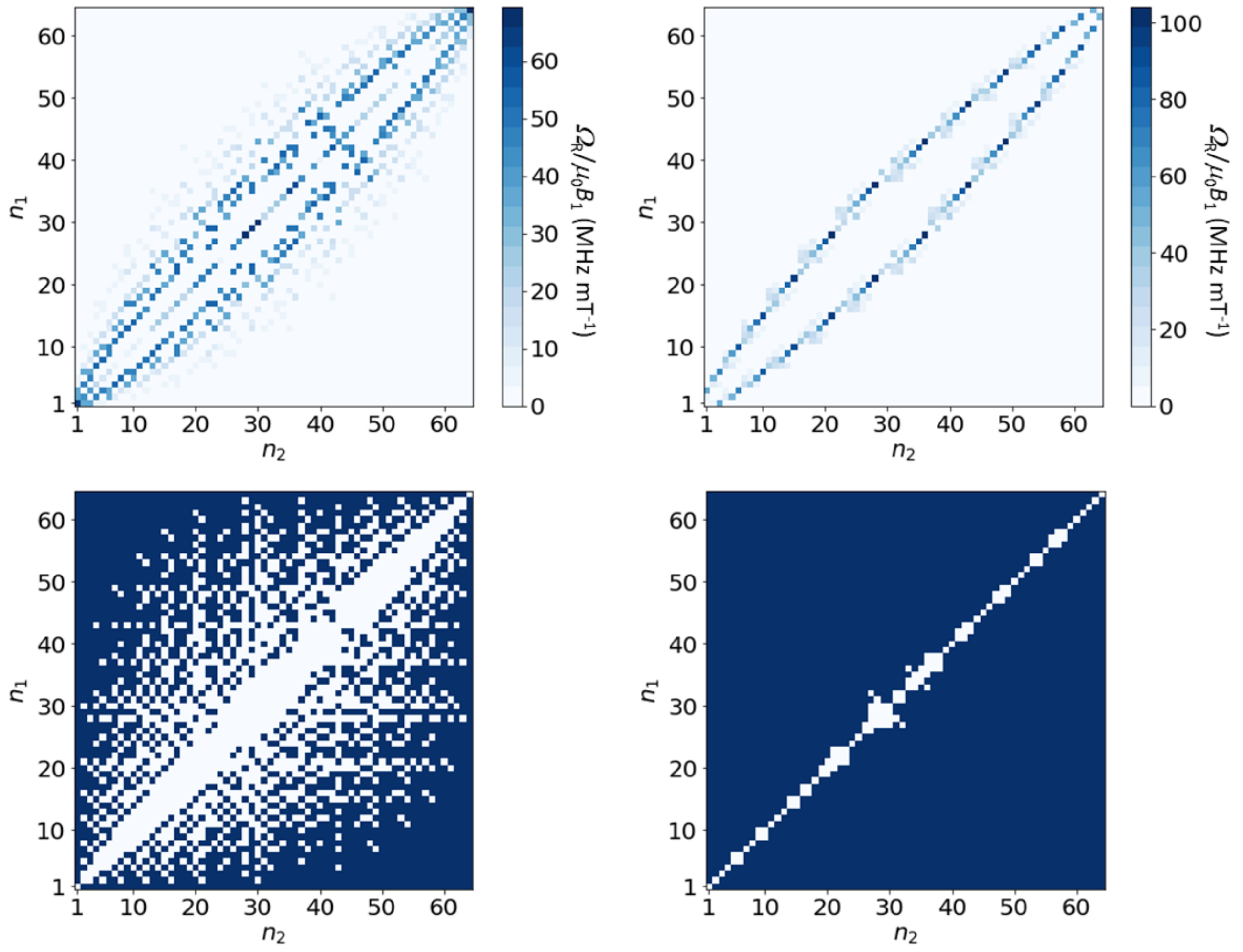

Supplementary Figure 5. Two methods to break universality in a pair of S = 7/2 qudits, to be compared with Fig. 7 of the main text. Top: Colour map of the Rabi frequencies calculated with Eq. (2) of the main text and the magnetic anisotropy constants of mmol L^{-1} for a 0.5 T magnetic field and J = 0, i.e. for uncoupled spins (left) and for a 10 T magnetic field and J = -0.42 GHz (right), i.e. for a magnetic field stronger than the magnetic anisotropy and the spin-spin coupling. Bottom: transitions (in blue dark) attainable by concatenating resonant transitions having Ω_R/B_1 > 0.2 MHz mT^{-1}. The white spots signal unfeasible gates.

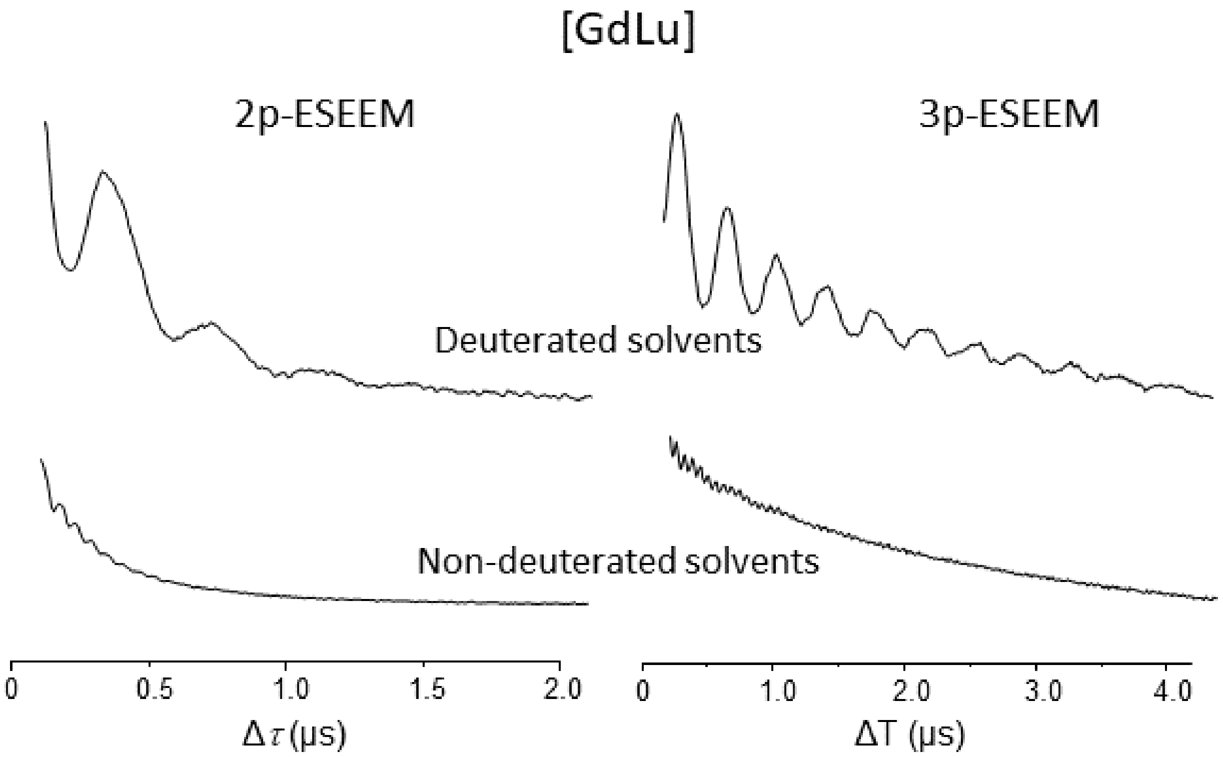

Supplementary Figure 6A. Representative 2-pulse (left, 400 mT) and 3-pulse (right, 410 mT) electron spin-echo decays for [GdLu] in solvents with different isotopic labelling. The main modulation, corresponding to the Larmor frequency of deuteron, disappears when solvents with natural isotopic hydrogen are used, and are then replaced with a proton Larmor frequency modulation. All data collected at 6 K on frozen solutions.

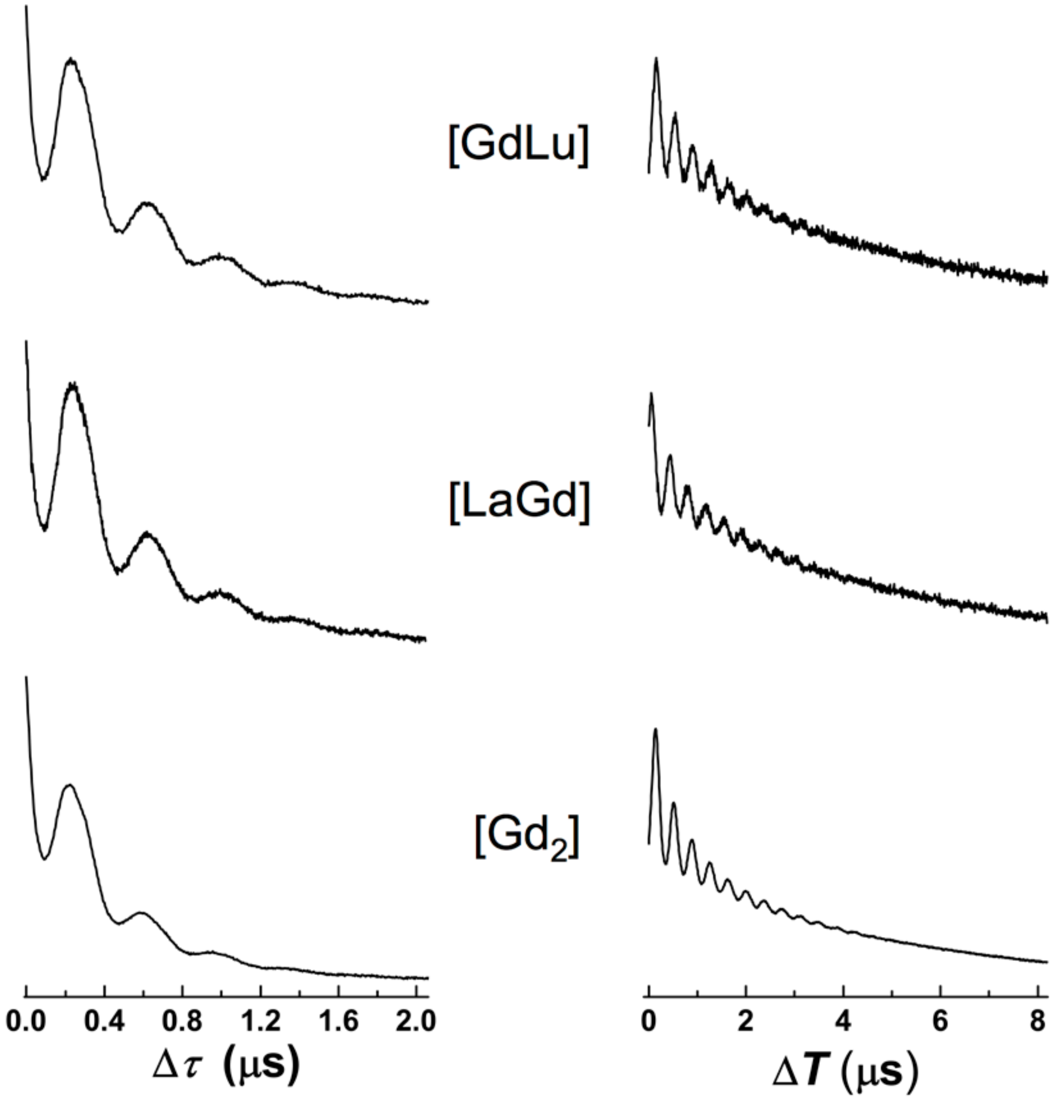

Supplementary Figure 6B. Representative 2-pulse (left, 400 mT) and 3-pulse (right, 410 mT) electron spin-echo decay for [LaGd], [GdLu] and [Gd_2] as indicated. All data collected at 6 K on frozen diluted MeOH-d^4:EtOH-d^6 solutions.

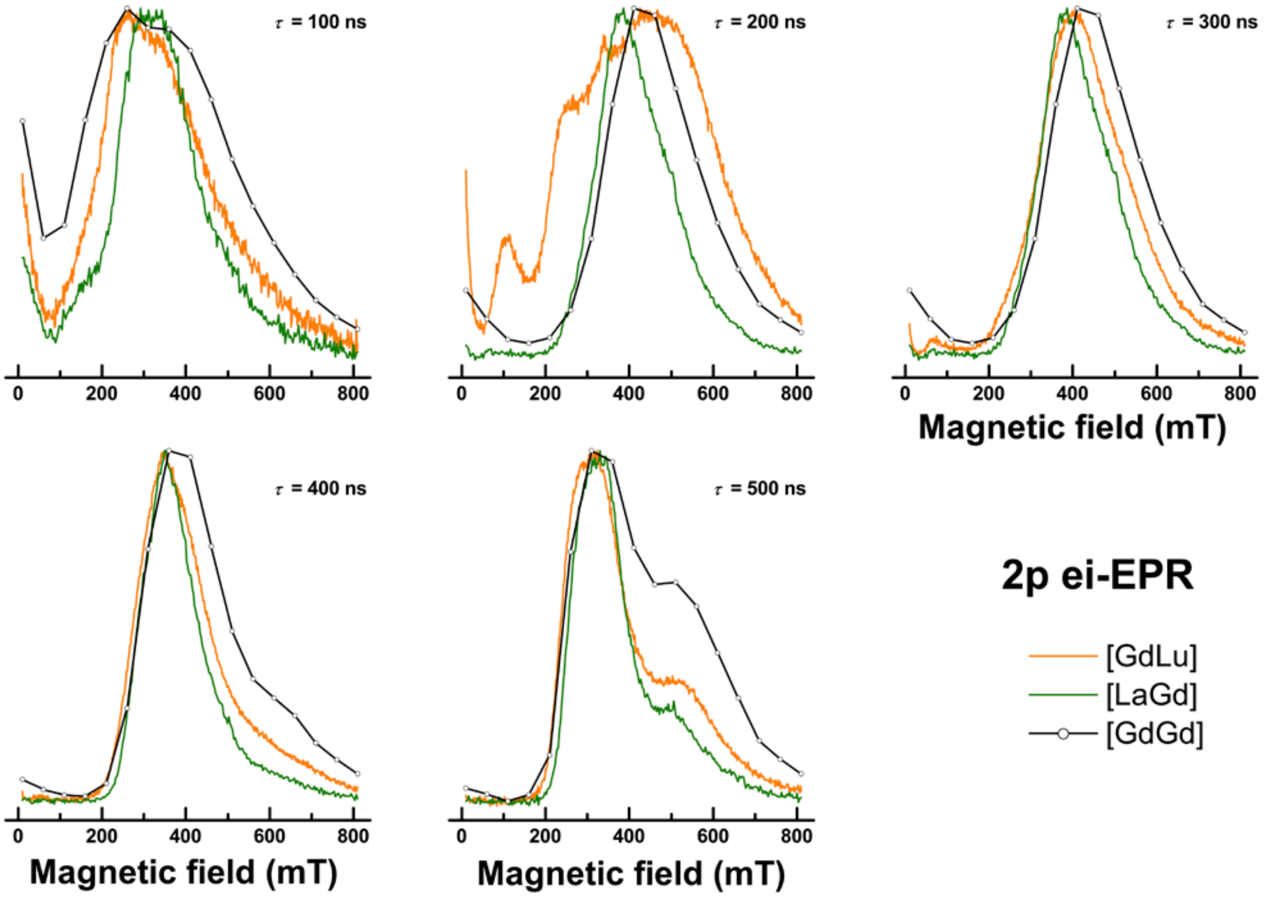

Supplementary Figure 7. Echo-induced EPR spectra for [LaGd], [GdLu] and [Gd_2] as derived from 2-pulse experiments (sequence $\pi/2 - \tau - \pi$) and varying τ, as indicated. All data collected at 6 K on frozen diluted MeOH-d^4:EtOH-d^6 solutions.

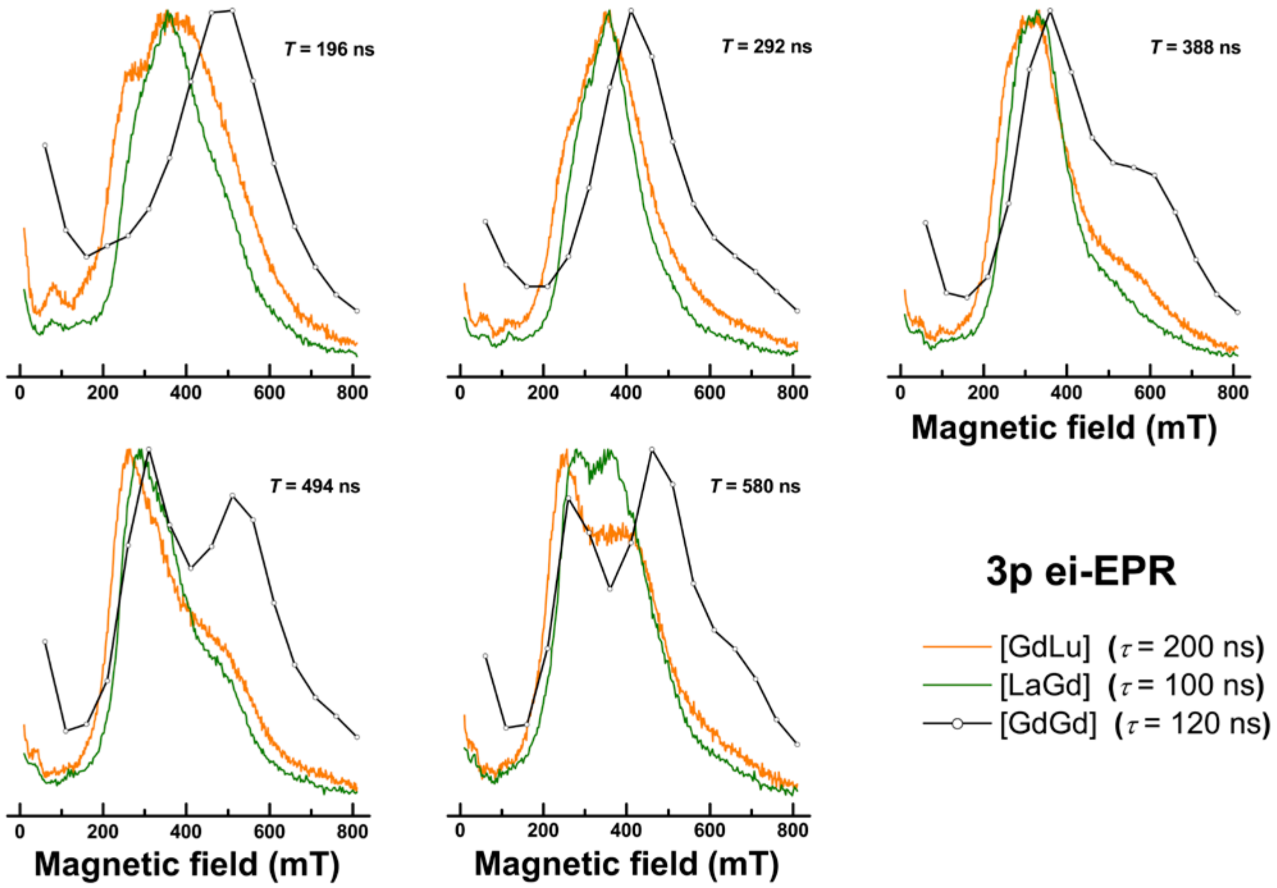

Supplementary Figure 8. Echo-induced EPR spectra for [LaGd], [GdLu] and [Gd_2] as derived from 3-pulse experiments (sequence $\pi/2$ – τ– $\pi/2$ – T – $\pi/2$) and varying T, as indicated. All data collected at 6 K on frozen diluted MeOH-d^4:EtOH-d^6 solutions.

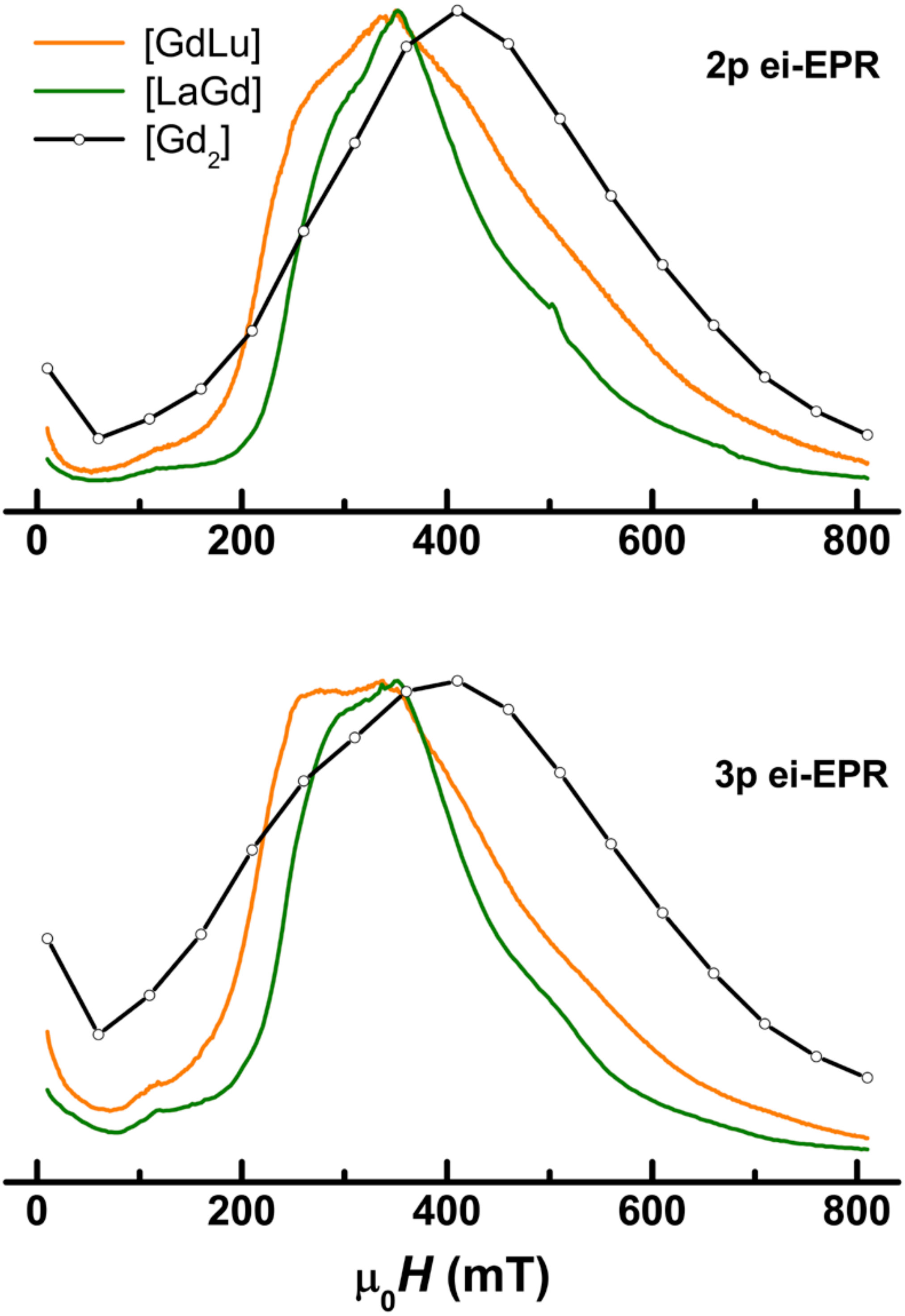

Supplementary Figure 9. Echo-induced EPR spectra for [LaGd], [GdLu] and [Gd_2] as derived from 2-pulse (top) and 3-pulse (bottom) experiments and summing for all τ and T, as indicated. All data collected at 6 K on frozen diluted MeOH-d^4:EtOH-d^6 solutions.

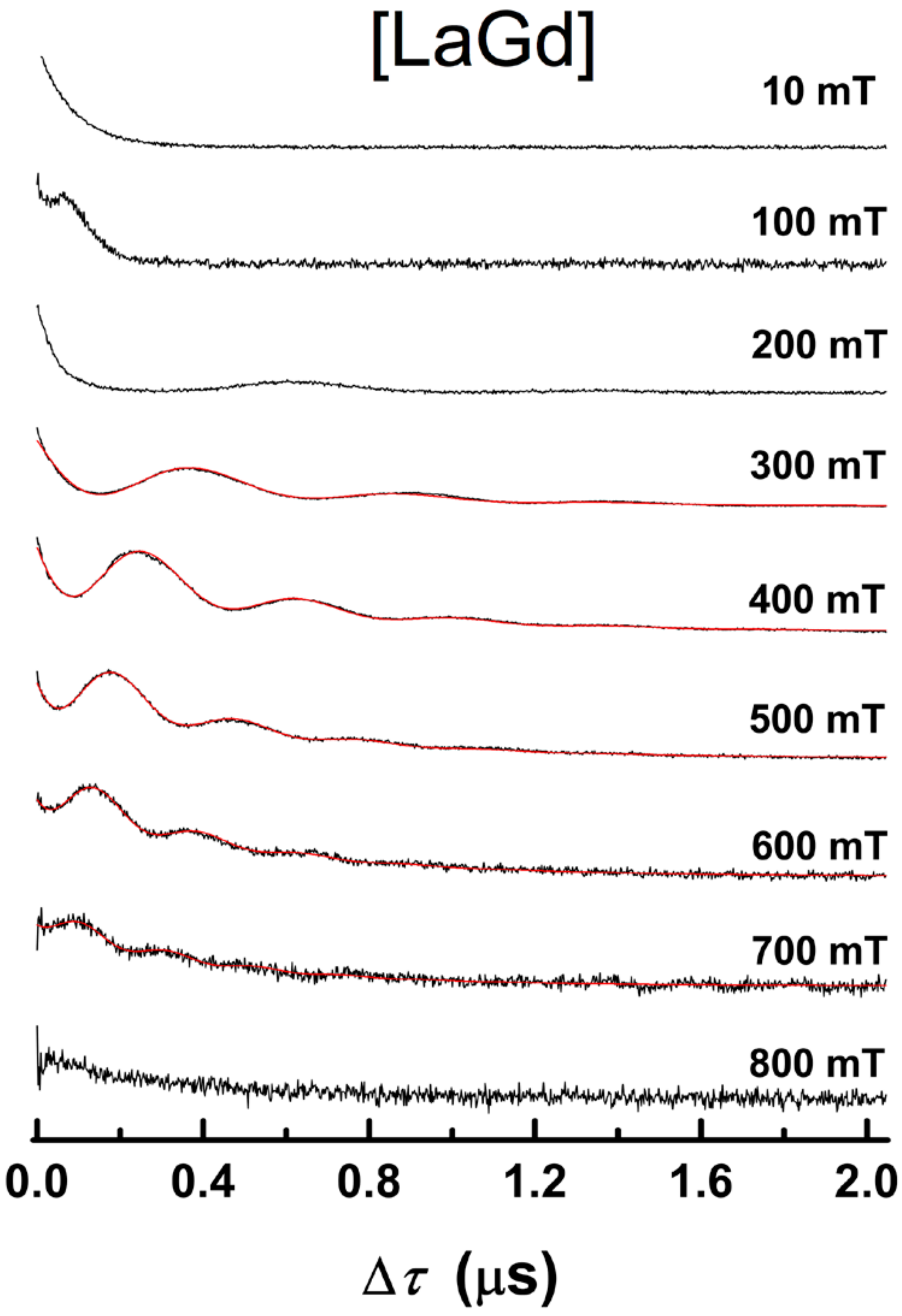

Supplementary Figure 10A. 2-pulse electron spin-echo decay at the indicated magnetic fields for [LaGd]. Full red lines correspond to the best-fit to the equation

$$y(\tau) = y_0 + A_{2p}e^{-2\tau/T_M}\{1 + ke^{-\lambda\tau}\cos(2\pi\nu\tau + \phi)\}$$

in which y_0 is background, A_{2p} the initial amplitude, T_M the phase memory time, k the relative amplitude of the modulated signal, λ the additional decay of the oscillating component and ν its frequency and ϕ the non-zero phase due to the detector dead-time. All data collected at 6 K on frozen diluted MeOH-d^4:EtOH-d^6 solutions.

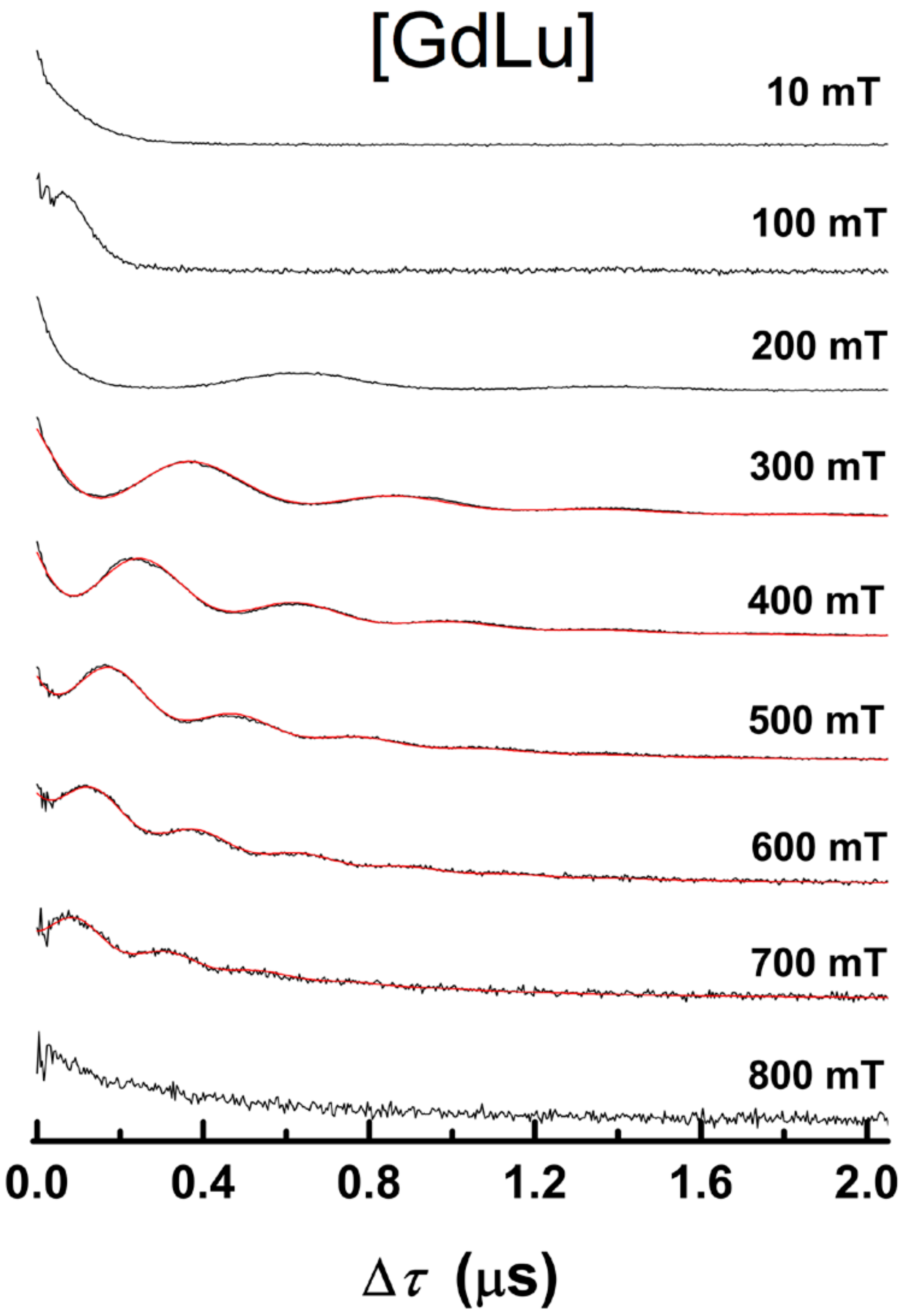

Supplementary Figure 10B. 2-pulse electron spin-echo decay at the indicated magnetic fields for [GdLu]. Full red lines correspond to the best-fit to the equation

$$y(\tau) = y_0 + A_{2p} e^{-2\tau/T_M}\{1 + k e^{-\lambda\tau}\cos(2\pi\nu\tau + \phi)\}$$

in which y_0 is background, A_{2p} the initial amplitude, T_M the phase memory time, k the relative amplitude of the modulated signal, λ the additional decay of the oscillating component and ν its frequency and ϕ the non-zero phase due to the detector dead-time. All data collected at 6 K on frozen diluted MeOH-d^4:EtOH-d^6 solutions.

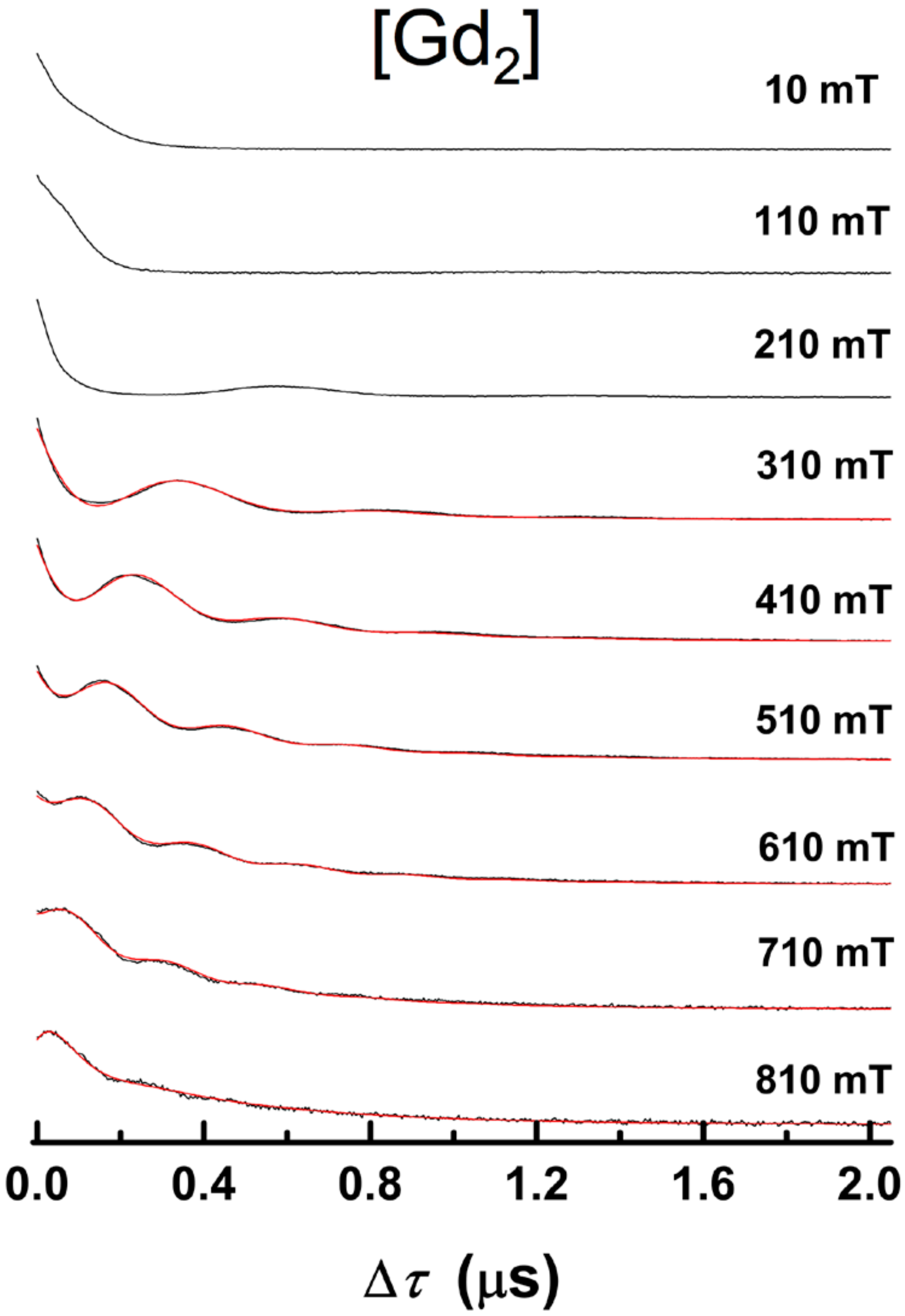

Supplementary Figure 10C. 2-pulse electron spin-echo decay at the indicated magnetic fields for [GdGd]. Full red lines correspond to the best-fit to the equation

$$y(\tau) = y_0 + A_{2p}e^{-2\tau/T_M}\{1 + ke^{-\lambda\tau}\cos(2\pi\nu\tau + \phi)\}$$

in which y_0 is background, A_{2p} the initial amplitude, T_M the phase memory time, k the relative amplitude of the modulated signal, λ the additional decay of the oscillating component and ν its frequency and ϕ the non-zero phase due to the detector dead-time. All data collected at 6 K on frozen diluted MeOH-d^4:EtOH-d^6 solutions.

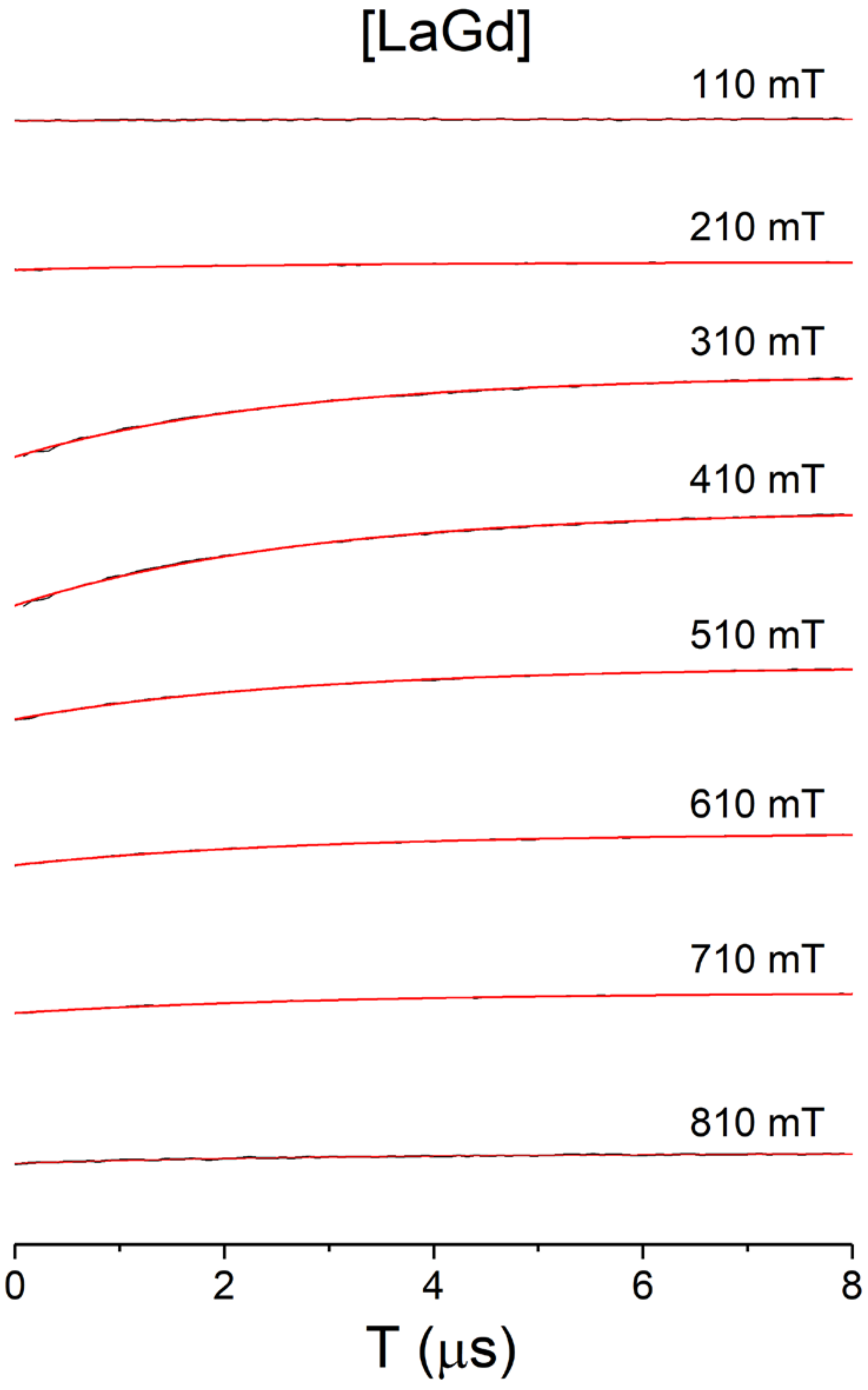

Supplementary Figure 11A. Measurements of the inversion recovery signal (sequence π – T – $\pi/2$ – τ – π) at the indicated magnetic fields for [LaGd]. Solid red lines correspond to the best-fit to the equation

$$y(\tau) = y_0 - A_{\mathrm{IR}} e^{-\mathrm{T}/\mathrm{T}_1}$$

in which y_0 is a background, A_{IR} the initial amplitude and T_1 the spin-relaxation time. All data collected at 6 K on frozen diluted MeOH-d^4:EtOH-d^6 solutions.

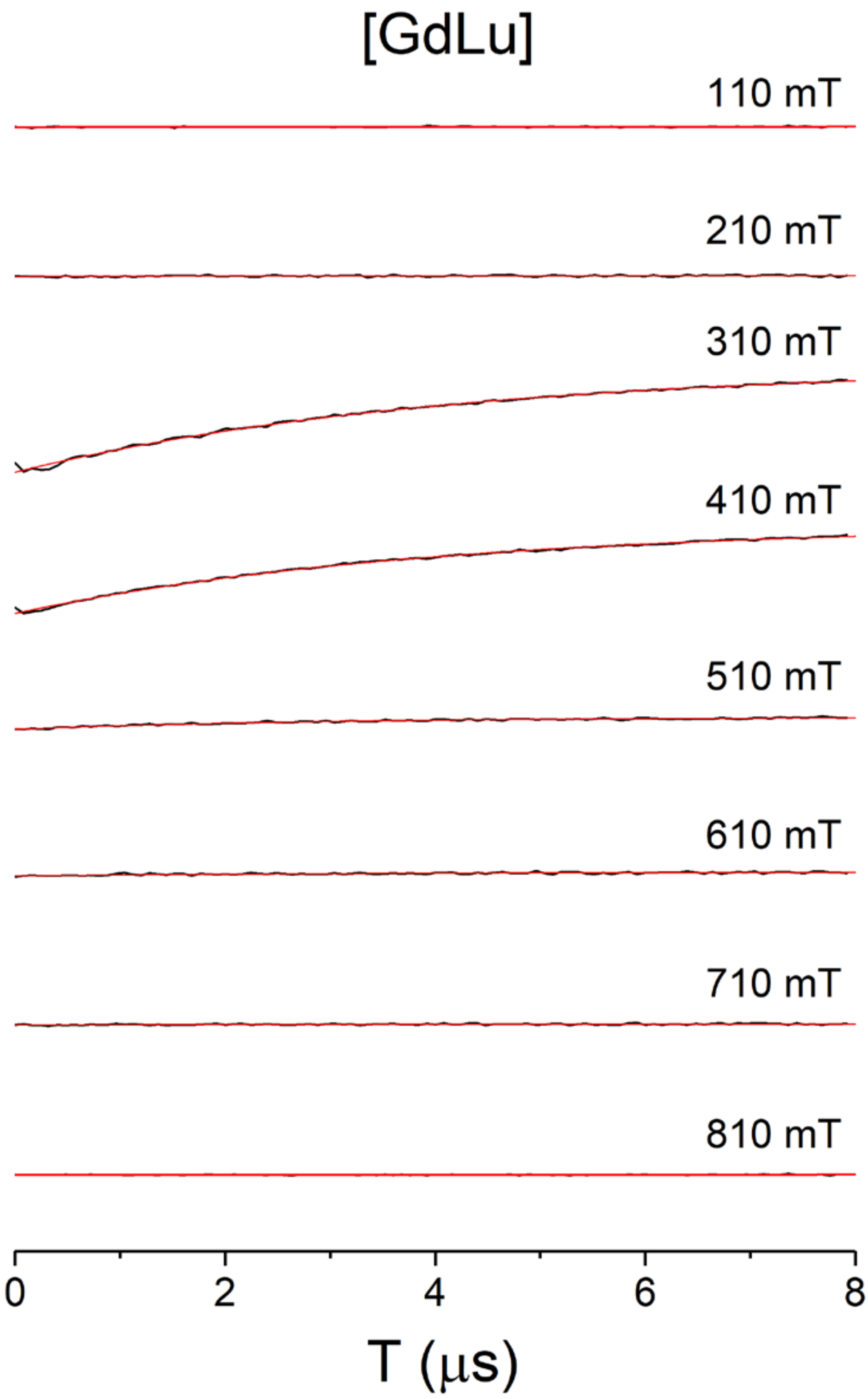

Supplementary Figure 11B. Measurements of the inversion recovery signal at the indicated magnetic fields for [GdLu]. Solid red lines correspond to the best-fit to the equation

$$y(\tau) = y_0 - A_{IR}e^{-T/T_1}$$

in which y_0 is a background, A_{IR} the initial amplitude and T_1 the spin-relaxation time. All data collected at 6 K on frozen diluted MeOH-d^4:EtOH-d^6 solutions.

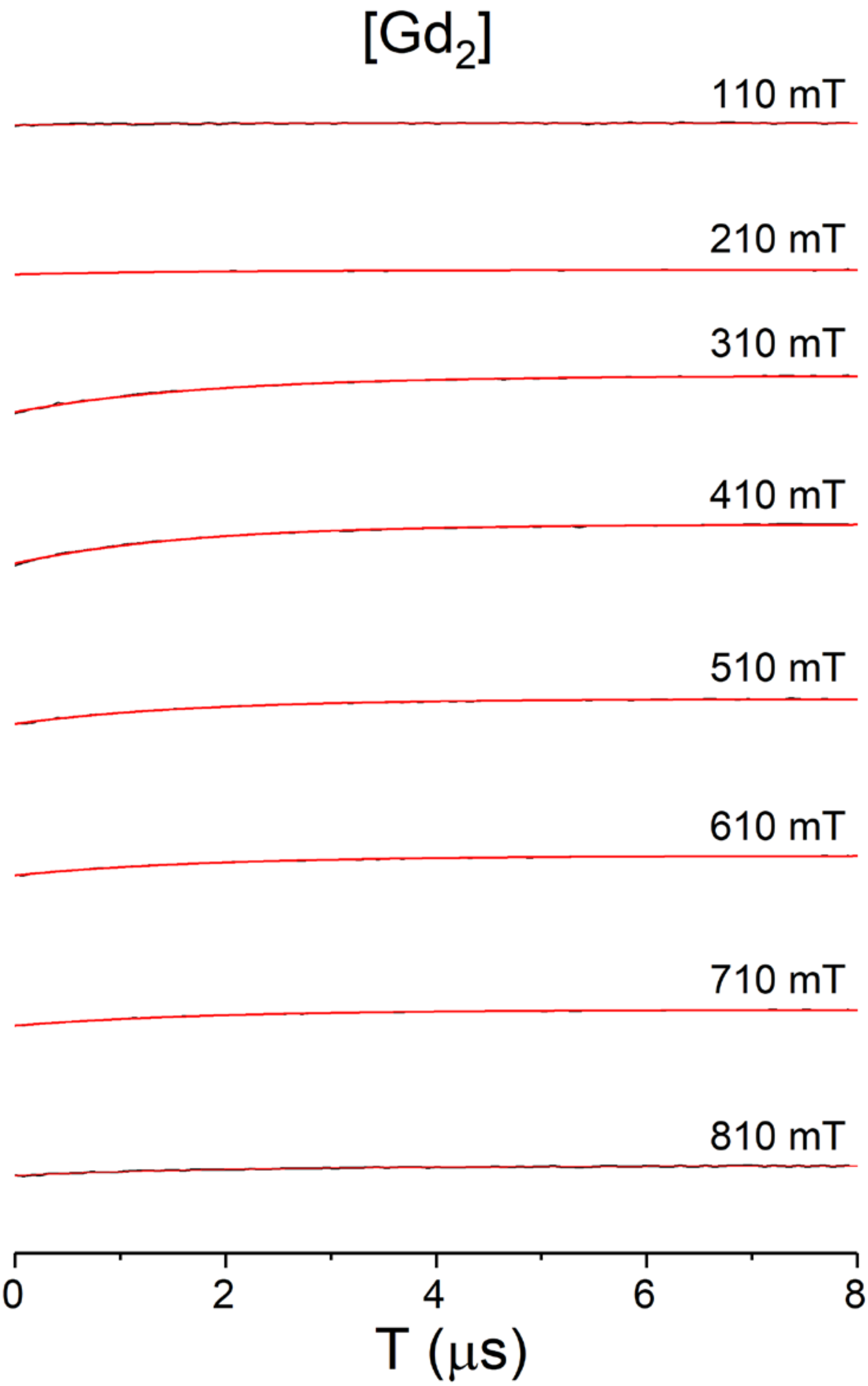

Supplementary Figure 11C. Measurements of the inversion recovery signal at the indicated magnetic fields for [Gd_2]. Solid red lines correspond to the best-fit to the equation

$$y(\tau) = y_0 - A_{IR}e^{-T/T_1}$$

in which y_0 is a background, A_{IR} the initial amplitude and T_1 the spin-relaxation time. All data collected at 6 K on frozen diluted MeOH-d^4:EtOH-d^6 solutions.

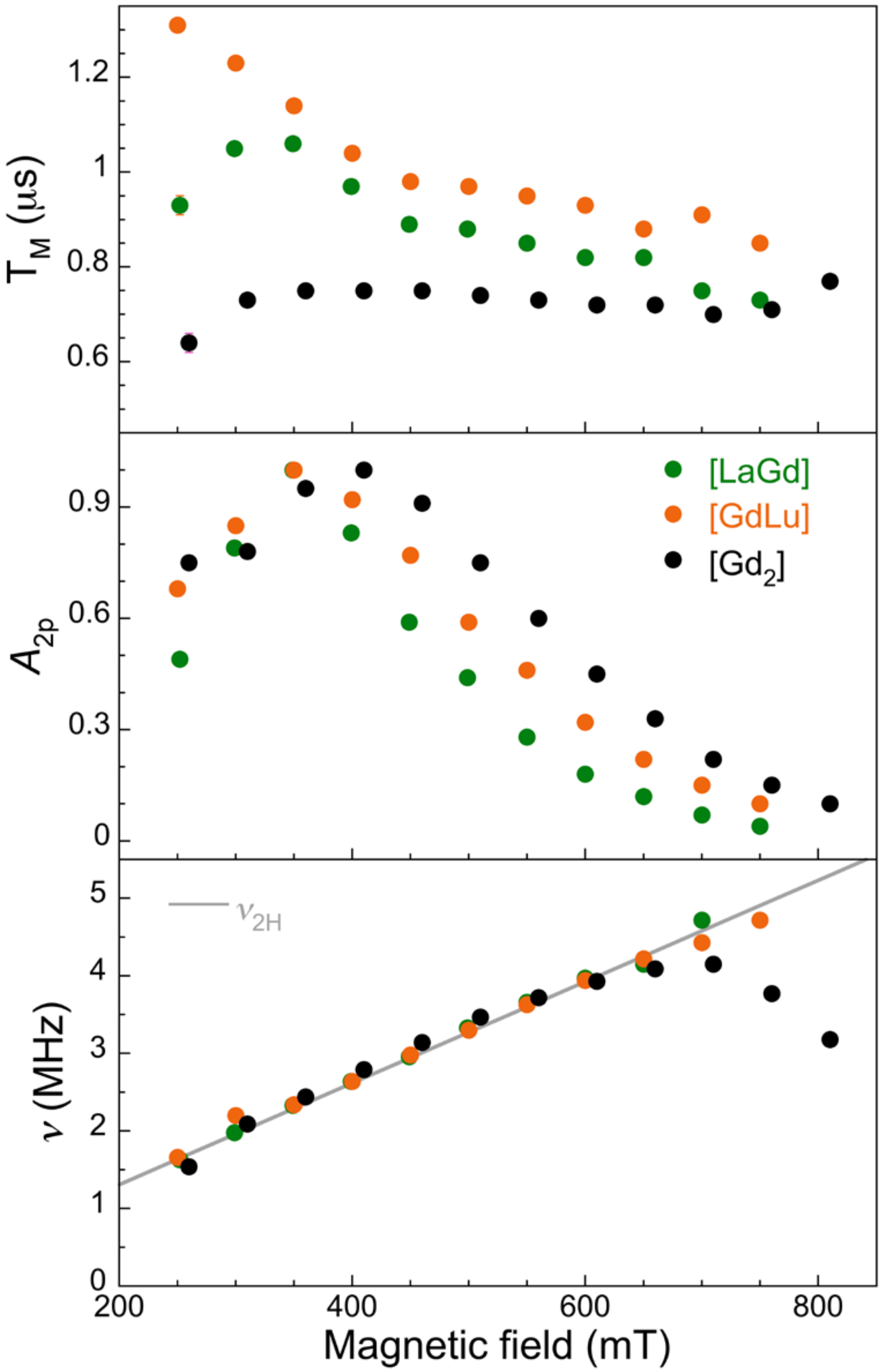

Supplementary Figure 12. Comparison of the field dependence of the phase memory time T_M (top), ESE amplitude (middle) and frequency of the modulation in the ESE decay ν (bottom) derived for diluted MeOH-d^4:EtOH-d^6 solutions of [LaGd], [GdLu] and [Gd_2] at 6 K from fits of 2-pulse ESE decays. The solid grey line (bottom) shows the field dependence of the ^2H nucleus Larmor frequency.

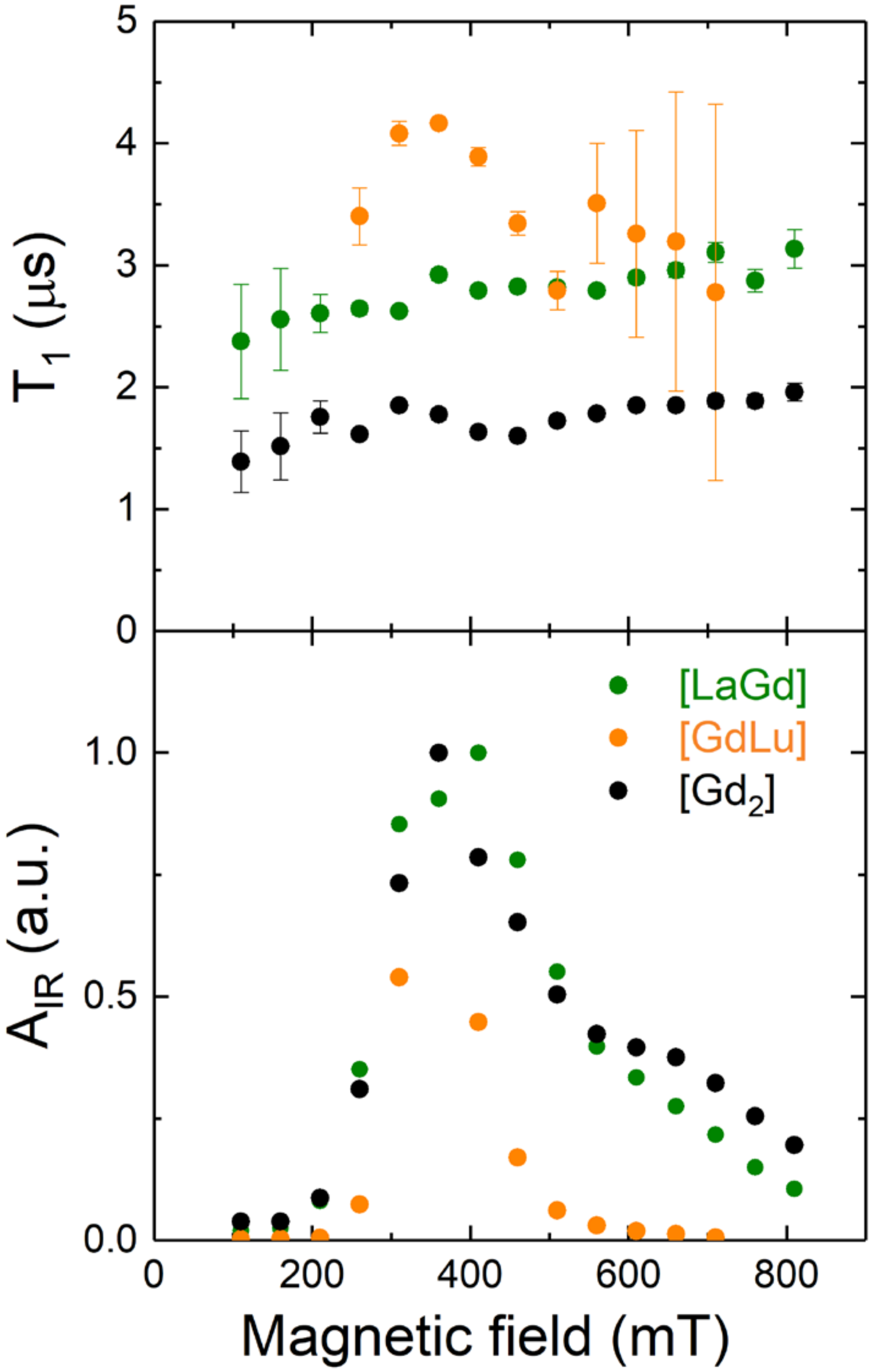

Supplementary Figure 13. Comparison of the field dependence of the spin-lattice relaxation T_1 (top) and the inversion recovery (bottom) derived for diluted MeOH-d^4:EtOH-d^6 solutions of [LaGd], [GdLu] and [Gd_2] at 6 K from fits of inversion recovery data.

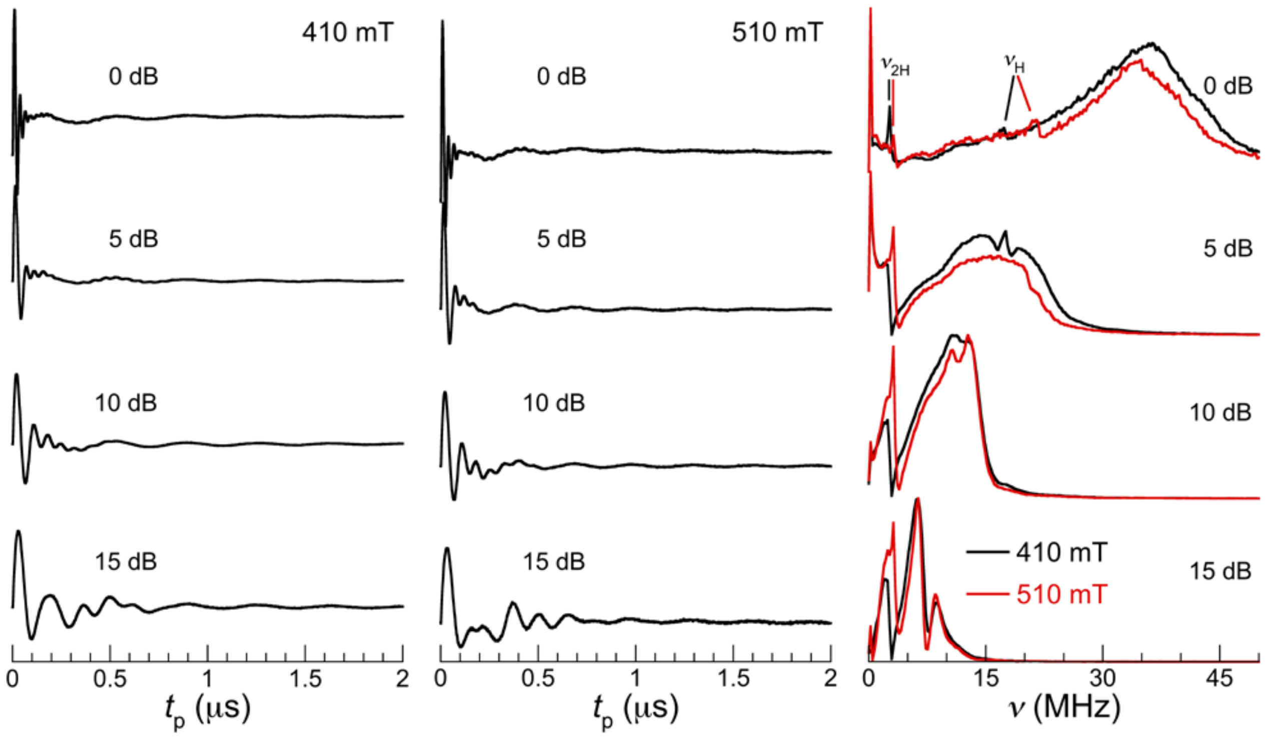

Supplementary Figure 14. Nutation experiments measured on a diluted MeOH-d^4:EtOH-d^6 solution of [LaGd] at 6 K and 410 (left) and 510 (middle) mT. Right: Normalised Fourier Transforms showing the main Rabi frequency and revealing additional oscillations with characteristic frequencies that correspond to the Larmor frequencies of ^{2}H and ^{1}H, as indicated.

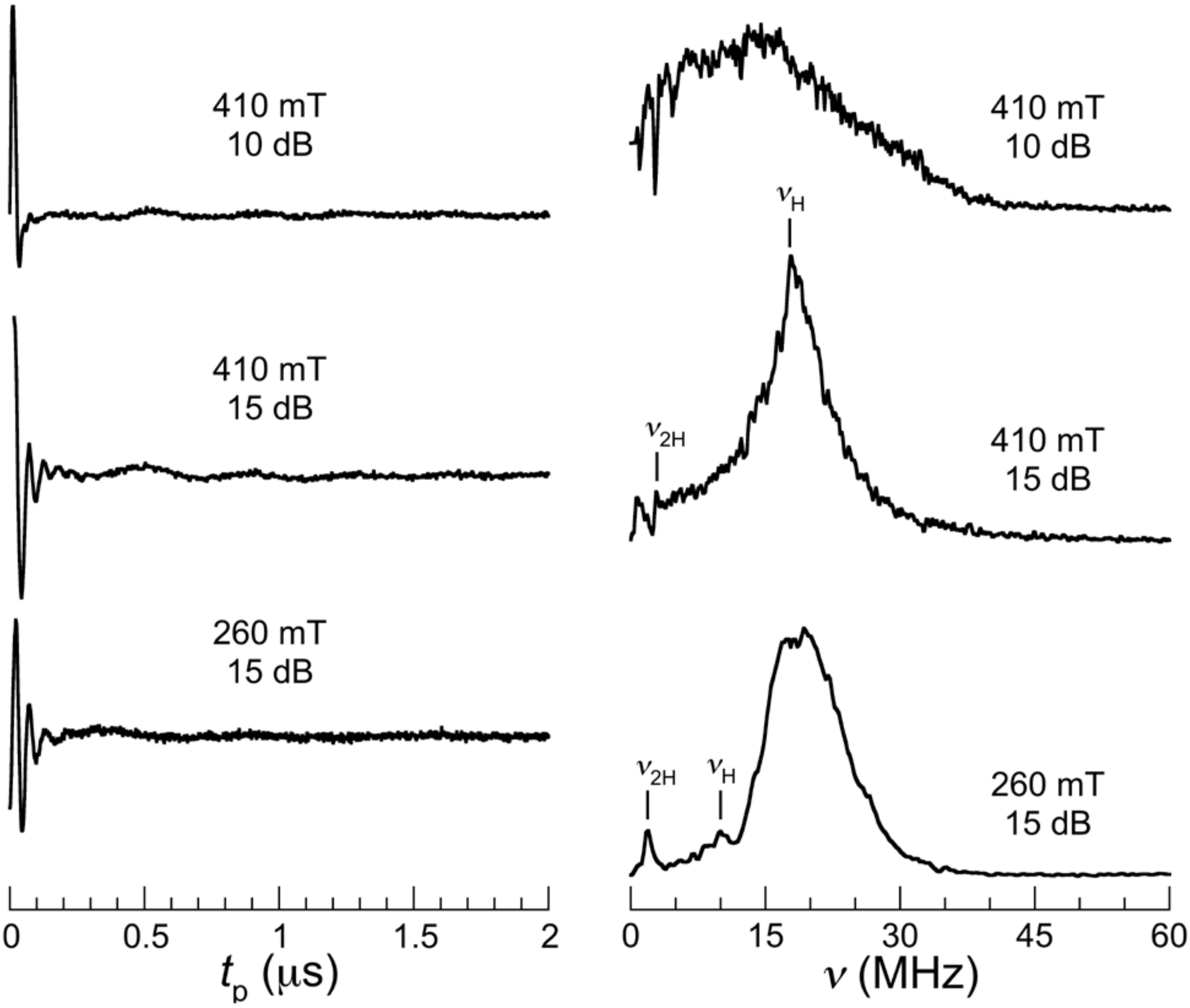

Supplementary Figure 15. Left: Nutation experiments measured on a diluted MeOH-d^4:EtOH-d^6 solution of [GdLu] at 6 K and 410 (10 and 15 dB) and 260 (15 dB) mT. Right: Corresponding Fourier Transforms showing the main Rabi frequency and revealing additional oscillations with characteristic frequencies that correspond to the Larmor frequencies of ^{2}H and ^{1}H, as indicated.

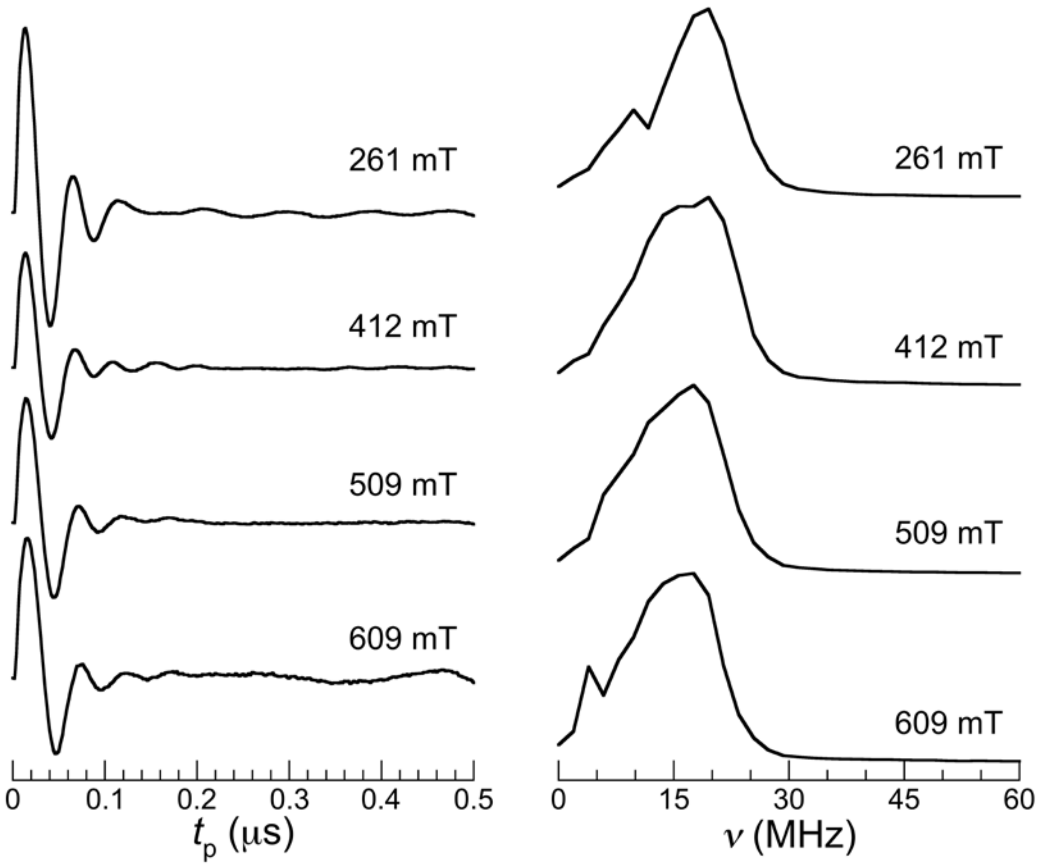

Supplementary Figure 16. Left: Nutation experiments measured on a diluted MeOH-d^4:EtOH-d^6 solution of [GdLu] at 6 K and increasing fields as indicated. Right: Corresponding Fourier Transforms showing the main Rabi frequency. Note that because these measurements were done with a lower resolution (256 points and a smaller interval of 2 ns), the frequency resolution is $\Delta\nu$ = 1.95 MHz, thus not allowing to observe with confidence the possible characteristic frequencies corresponding to the Larmor frequencies of ^2H and ^1H.
