# Supplementary material for: A dissymmetric [Gd2] coordination molecular dimer hosting six addressable spin qubits
Source: Commun Chem. 2020 Nov 20;3:176. doi: 10.1038/s42004-020-00422-w (PMC9814487; doi:10.1038/s42004-020-00422-w)
Supplement: Supplementary file 2 — Description of Additional Supplementary Files [file 42004_2020_422_MOESM2_ESM.pdf]

Description of Additional Supplementary Files

File Name: Supplementary Data 1
Description: X-ray crystallographic coordinates for the structure of complex [LaGd]

File Name: Supplementary Data 2
Description: X-ray crystallographic coordinates for the structure of complex [GdLu]
